# Supplementary material for: Tissue Usage Preference and Intrinsically Disordered Region Remodeling of Alternative Splicing Derived Proteoforms in the Heart
Source: J Proteome Res. 2024 Mar 8;23(8):3161–73. doi: 10.1021/acs.jproteome.3c00789 (PMC11296937; doi:10.1021/acs.jproteome.3c00789)
Supplement: Supplementary file 1 — pr3c00789_si_001.pdf [file pr3c00789_si_001.pdf]

Supporting Information

Tissue usage preference and intrinsically disordered region remodeling of alternative splicing derived proteoforms in the heart

[1]Boomathi Pandi, [1]Stella Brenman, [1]Alexander Black, [1]Dominic C. M. Ng, [1,3]Edward Lau, [1,2,3,*]Maggie P. Y. Lam

[1] Department of Medicine/Division of Cardiology
[2] Department of Biochemistry & Molecular Genetics
[3] Consortium for Fibrosis Research and Translation (CFReT)
University of Colorado School of Medicine
Aurora, CO 80045, USA

* Correspondence: Maggie P. Y. Lam, PhD
maggie.lam@cuanschutz.edu

Table of Contents

Supplemental Figures

Figure S1: PepQuery2 peptide-spectrum matches for two undocumented tensin-1 -J1 isoform peptides.

Figure S2: AlphaFold2 proposed structure and flDPnn predicted sequence disorders and functional features for the PDLIM3-3 isoform.

Figure S3: AlphaFold2 proposed structure and flDPnn predicted sequence disorders and functional features for the PDLIM5 sequence and the -3 and -6 alternative isoforms.

Figure S4: Proportion of residues within flDPnn predicted IDRs that are associated with A. protein binding or B. RNA binding function.

Figure S5: Alternative isoforms with deleted variant regions from canonical sequences

show evidence of IDR remodeling.

Supplemental Tables

Table S1: limma results from canonical database search using MSFragger/Philosopher in PXD008722.

Table S2: limma results from JCAST canonical + isoform database search using MSFragger/Philosopher in PXD008722.

Table S3: limma results for alternative isoforms quantified in validation data.

Table S4: All identified isoforms by Comet/Percolator and MSFragger/Philosopher.

Table S5: Annotated protein features in identified isoforms.

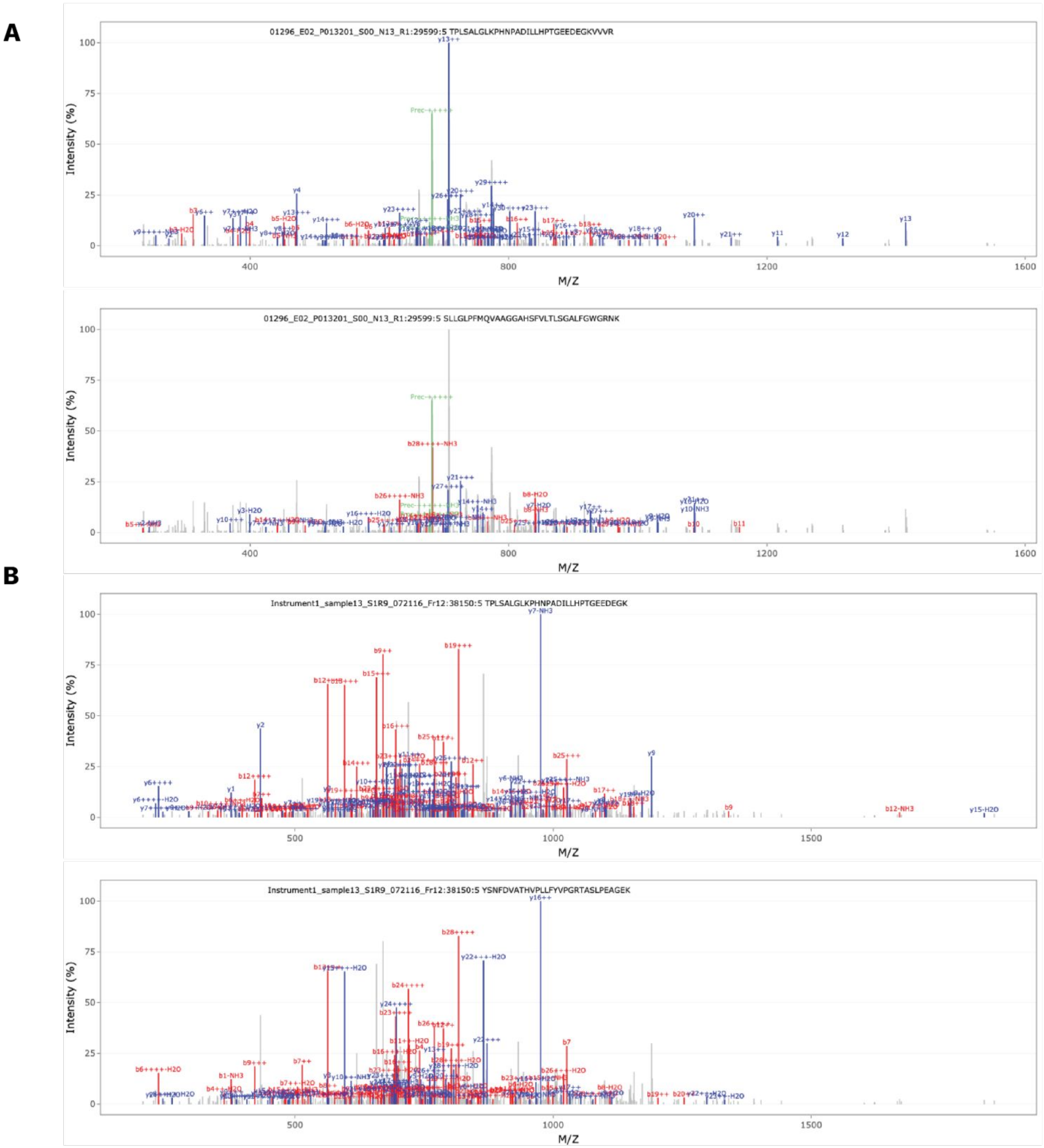

Supplemental Figure S1: PepQuery2 peptide-spectrum matches for two undocumented tensin-1 -J1 isoform peptides. **A.** Peptide spectrum match of the splice junction peptide TPLSALGLKPHNPADILLHPTGEEDEGKVVVR against the “29_healthy_human_tissues” (PXD010154) dataset (hyperscore 83.29) (top) and the corresponding peptide spectrum match from the canonical database search (bottom). **B.** Peptide spectrum match of the splice junction

peptide TPLSALGLKPHNPADILLHPTGEEDEGK against the "GTEx_32_Tissues_Proteome" (PXD016999) dataset (hyperscore 99.63) (top) and the corresponding peptide spectrum match from the canonical database search (bottom).

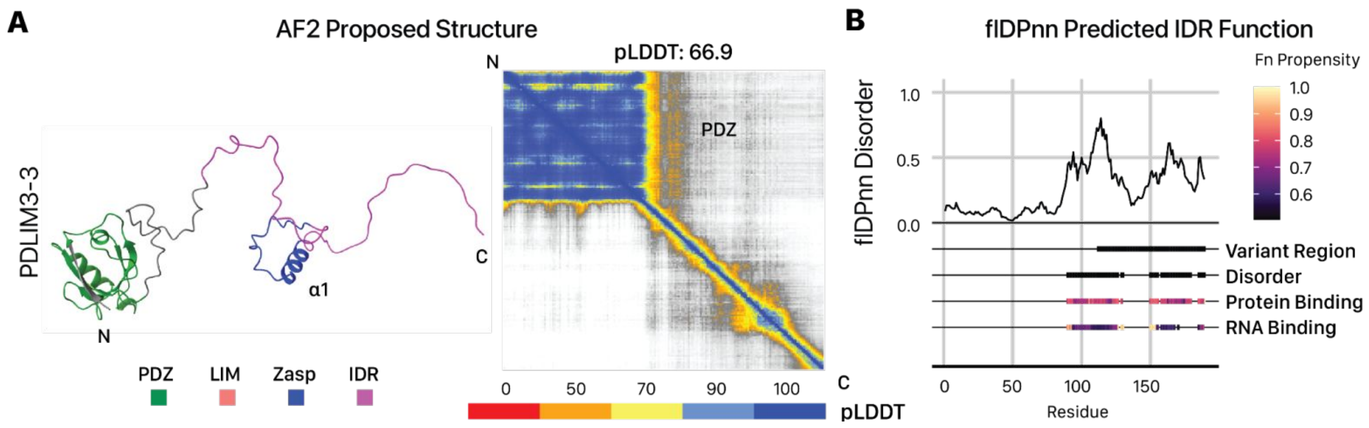

Supplemental Figure S2: A. AlphaFold2 proposed structure and **B.** fIDPnn predicted sequence disorders and functional features for the PDLIM3-3 isoform. See legends for Figure 2 for additional details.

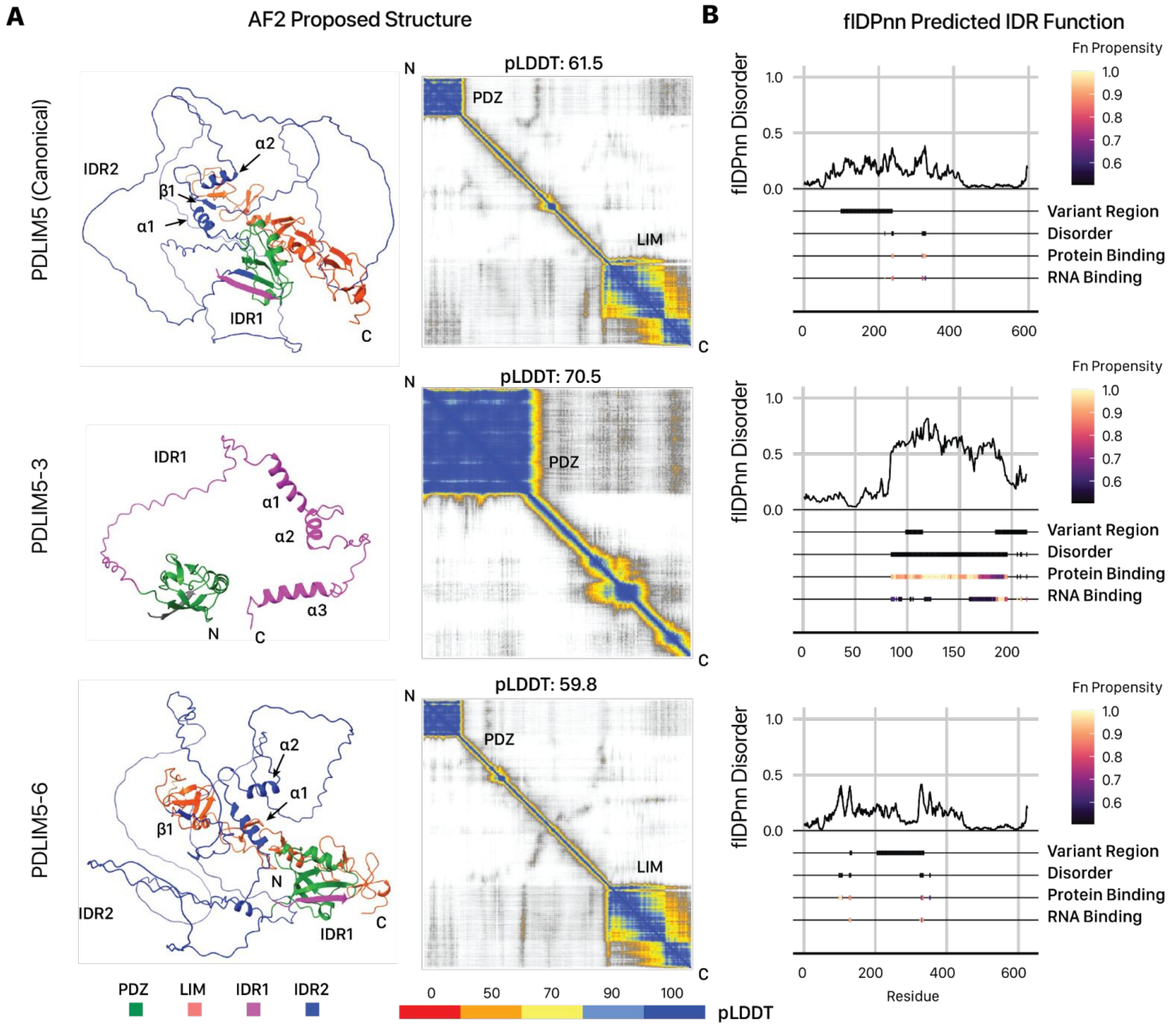

Supplemental Figure S3: A. AlphaFold2 proposed structure and **B.** fIDPnn predicted sequence disorders and functional features for the PDLIM5 sequence and the -3 and -6 alternative isoforms. See legends for Figure 2 for additional details.

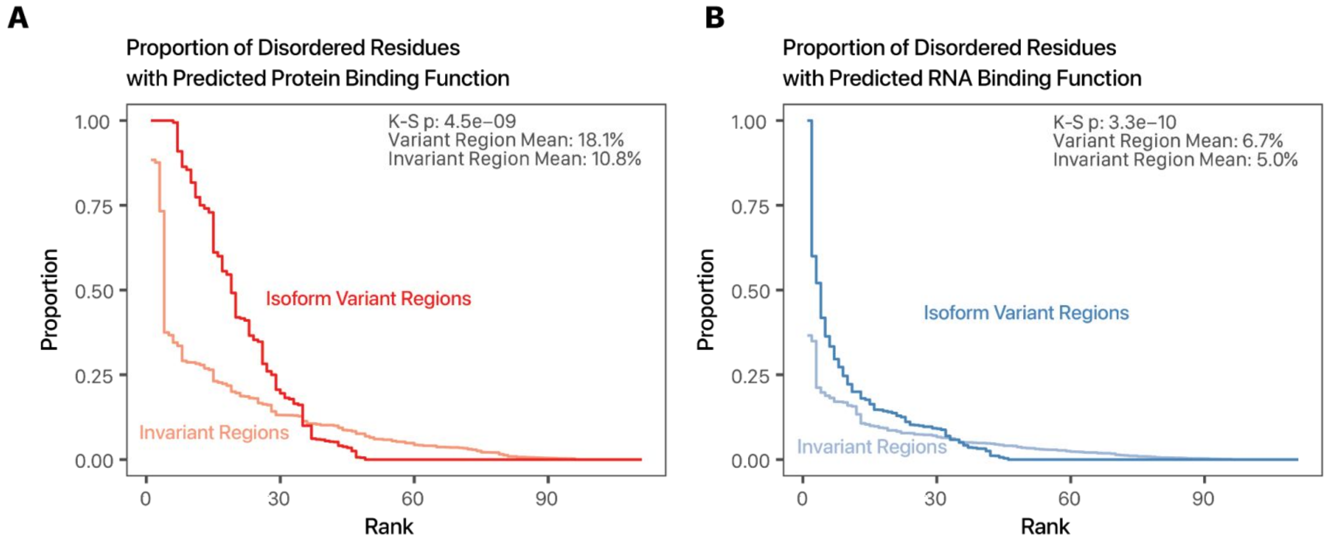

Supplemental Figure S4: Proportion of residues within flDPnn predicted IDRs that are associated with **A.** protein binding or **B.** RNA binding function. K-S: two-sample Kolmogorov-Smirnov test. Darker line: IDR residues within isoform variant regions; lighter line: outside the isoform variant regions.

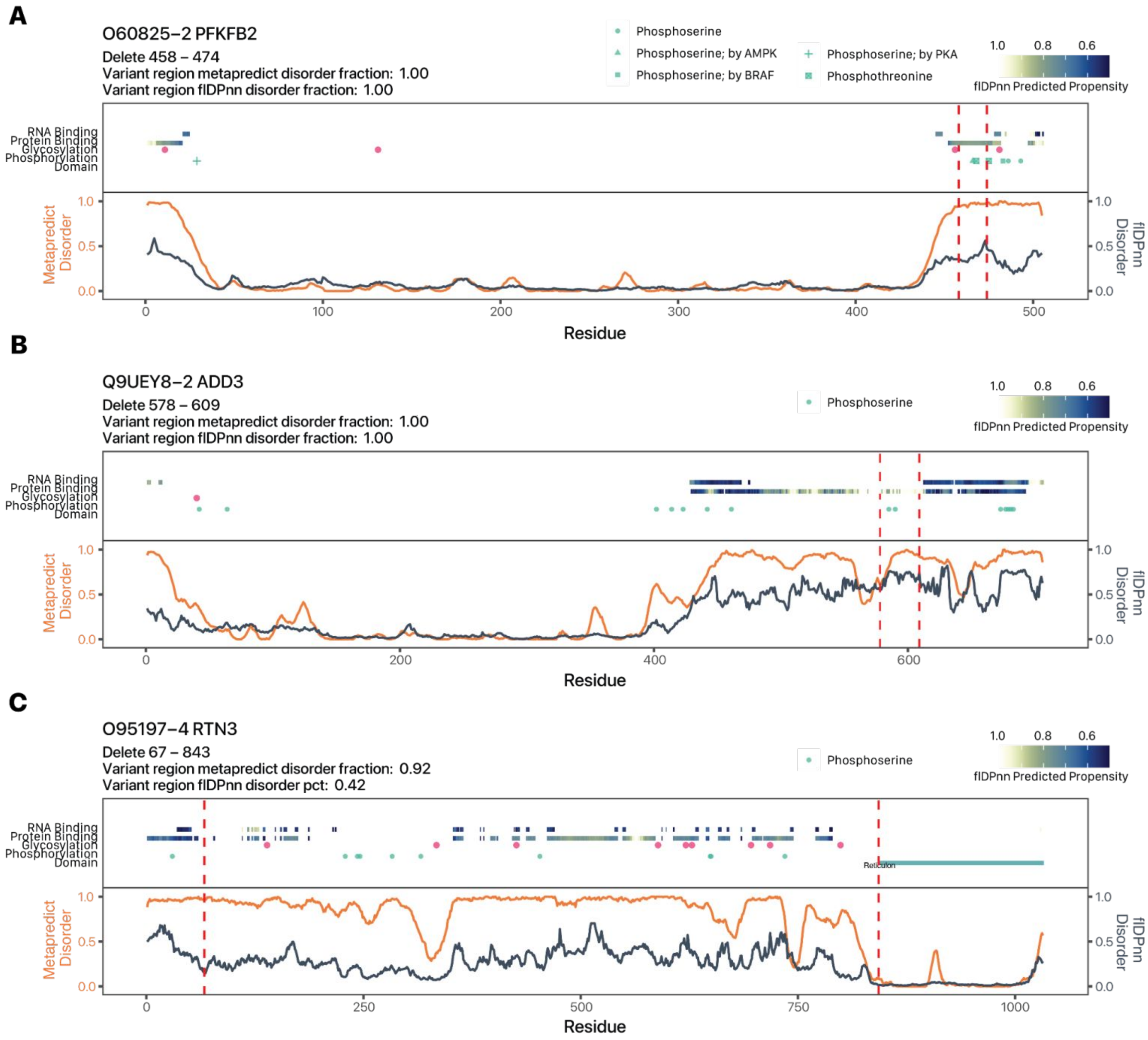

Supplemental Figure S5: Alternative isoforms with deleted variant regions from canonical sequences show evidence of IDR remodeling. Three isoforms with variant regions removed from the canonical sequences are shown: **A.** PFKFB2-2; **B.** ADD3-2; **C.** RTN3-4. See legends for Figure 5 for additional details.
